# From Chronic Alcohol Consumption to Coma: Report of an Uncommon Cause

**DOI:** 10.7759/cureus.36411

**Published:** 2023-03-20

**Authors:** João Pedro Melo e Silva, Antony Soares Dionísio, Daniela Barbosa Mateus, Filipa Pais Silva, João Gonçalves Pereira

**Affiliations:** 1 Intensive Care Unit Department, Hospital de Vila Franca de Xira, Vila Franca de Xira, PRT; 2 Internal Medicine Department, Centro Hospitalar de Lisboa Ocidental - Hospital São Francisco Xavier, Lisboa, PRT; 3 Internal Medicine Department, Hospital de Vila Franca de Xira, Vila Franca de Xira, PRT

**Keywords:** marchiafava-bignami disease, thiamine deficiency, demyelination, corpus callosum, coma, alcoholism

## Abstract

Marchiafava-Bignami disease is a rare condition characterized by demyelination of the corpus callosum that can evolve into necrosis. It is associated with thiamine deficiency, chronic alcohol consumption, and less frequently, severe malnutrition. The diagnosis is based on clinical presentation - altered mental state and changes in a neurological examination - and on neuroimaging studies, especially magnetic resonance imaging. Treatment with parenteral thiamine is recommended.

The authors present a case of a 50-year-old male, with chronic alcohol abuse and malnutrition, admitted to the hospital with an acute form of the Marchiafava-Bignami disease. An early diagnosis and treatment facilitated neurological and cognitive recovery.

## Introduction

Marchiafava-Bignami disease (MBD) is a rare condition, related to thiamine deficiency, mostly present in chronic alcohol consumers. It is sometimes associated with severe malnutrition, even without a history of severe alcohol intake [1,2]. It is characterized by demyelination of the corpus callosum, which can evolve into necrosis [1,3]. The clinical presentation often includes an altered mental state, namely confusion, impaired memory, disorientation, delirium, or even unconsciousness. Impaired walking, dysarthria, mutism, dysphagia, signs of interhemispheric disconnection or split-brain syndrome, pyramidal signs, primitive reflexes, rigidity, incontinence, sensory symptoms, gaze palsy or diplopia, seizures, and coma are clinical signs that may be found in MBD patients [1,3].

Before the advancements in imaging techniques, the diagnosis of MBD was done almost exclusively by autopsy. Nowadays, advanced neuroimaging, such as magnetic resonance imaging (MRI), allows clinicians to achieve an early diagnosis, through an accurate evaluation of brain involvement. The proposed treatment of MBD consists of the administration of high doses of thiamine and corticosteroids [1,4]. However, since this is a rare condition, there are no trials to support the use of those treatments [5]. The described outcomes are usually poor, with almost 90% of coma patients with a bad outcome, although some patients achieved complete recovery after weeks or months of treatment [3,6].

## Case presentation

A 50-year-old male presented to the emergency room (ER) due to acute loss of consciousness, lasting for 12 hours. His relatives disclosed a history of tremors, dizziness, and confusion in the days prior to hospital admission. A history of chronic alcohol and tobacco abuse was disclosed. He had a cranial prosthesis, a consequence of severe head trauma at the age of three. Malnourishment was evident. He presented in coma (Glasgow Coma Scale score of 3); his pupils were isochoric and isoreactive to light but with conjugate gaze deviation; fasciculations of the lower limbs were noted. He also presented bradycardia (heart rate 32 bpm), hypotension (arterial pressure 83/39 mmHg), and hypothermia (temperature 34.1ºC).

Laboratory findings (Table 1) revealed metabolic acidemia, thrombocytopenia, and hypokalemia. Urine screening was positive for benzodiazepines. An electrocardiogram showed sinus bradycardia.

**Table 1:** Laboratory findings on the day of admission to the ER. ER: emergency room

Blood Workup			
Test	Value	Reference Range	Comment
Hemoglobin	13.9 g/dL	13.0-16.9 g/dL	Normal
White Blood Cells	4200/µL	4000-11000/µL	Normal
Platelets	119,000/µL	150-400/µL	Below Normal
C Reactive Protein	0.56 mg/dL	Less than 0.5 mg/L	Above Normal
Glucose	87 mg/dL	70-140 mg/dL	Normal
Creatinine	0.59 mg/dL	0.7-1.3 mg/dL	Below Normal
Sodium	144 mmol/L	135-146 mmol/L	Normal
Potassium	2.84 mmol/L	3.5-4.5 mmol/L	Below Normal
Total Bilirubin	0.42 mg/dL	0.1-1.2 mg/dL	Normal
Alanine Transaminase	79 U/L	Less than 46 U/L	Above Normal
Aspartate Transaminase	110 U/L	Less than 50 U/L	Above Normal
Thyroid-Stimulating Hormone	1.3 mU/L	0.4-4.2 mU/L	Normal
Free T4	1.1 ng/dL	0.9-2.3 ng/dL	Normal
Folic acid	1.1 ng/mL	2.7 to 17.0 ng/mL	Below normal
B12 vitamin	1761 pg/mL	160 to 950 pg/mL	Above normal
Arterial blood gases			
pH	7.27	7.35-7.45	Below normal
pO2	110.4	More than 65	Normal
pCO2	41.7	35-45	Normal
HCO3	18.6	22-26	Below normal
Urinary test for Drugs	Positive for benzodiazepines. Negative to other drugs		
Human Immunodeficiency Virus (HIV) antigen test	Negative		
Cerebrospinal fluid	Normal		

**Figure 1:**
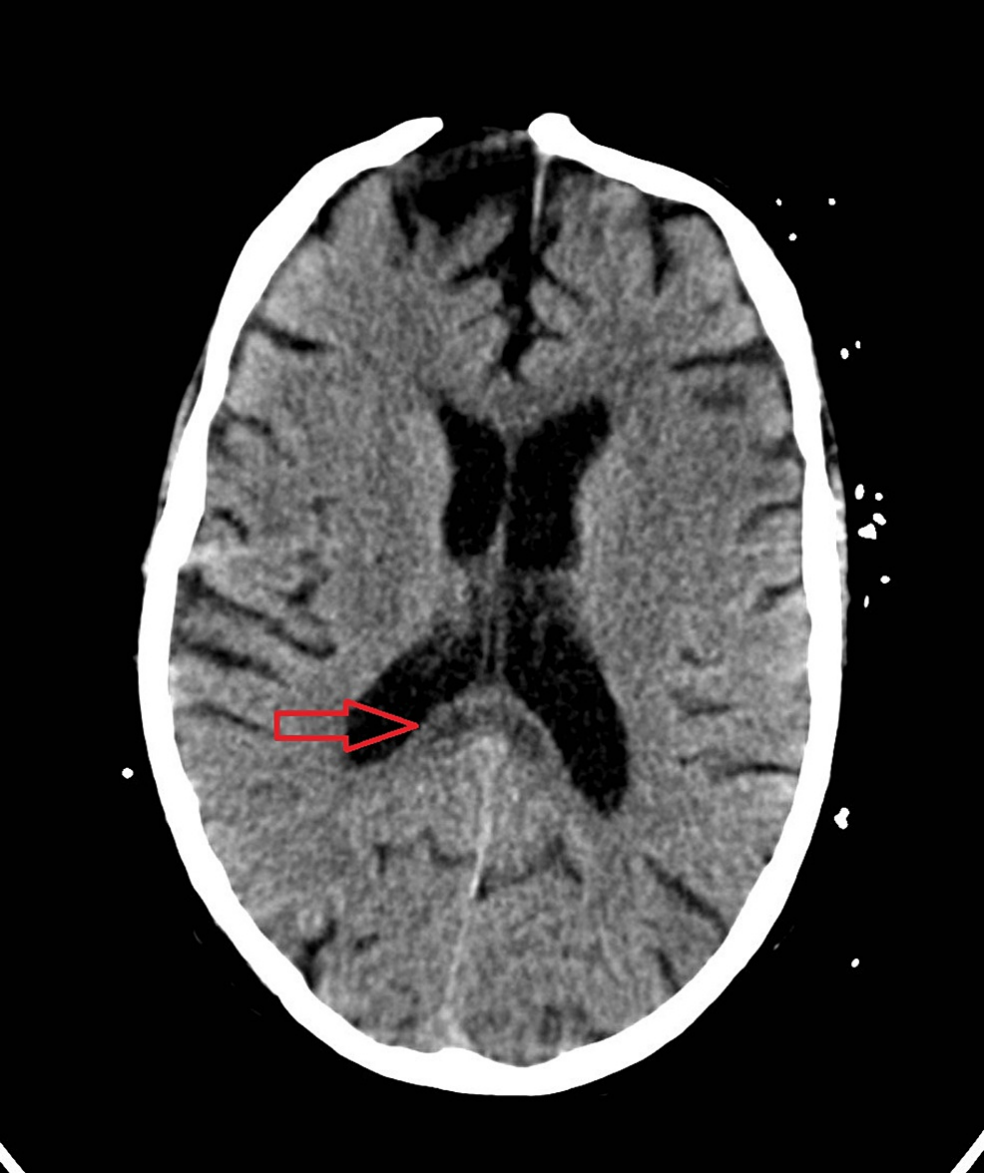
CT scan showing lesion in the splenium of the corpus callosum (arrow). Evidence of bone discontinuity and bilateral anterior frontal cortico-subcortical encephalomalacia. CT: computed tomography

A cranial computed tomography unveiled unspecific rounded areas with hypodensity in the splenium and genu of the corpus callosum. Signs of a previous surgical approach in the anterior frontal region, with evidence of bone discontinuity and bilateral anterior frontal cortico-subcortical encephalomalacia, were also present (Figure 1).

Flumazenil, tiapride, thiamine, and levetiracetam were all administered with no significant clinical improvement.

The patient was admitted to the Intensive Care Unit (ICU). Intubation and invasive mechanical ventilation were started. Administration of vasopressors ensured a mean blood pressure above 60 mmHg. A lumbar puncture unveiled an analytically normal cerebrospinal fluid. An electroencephalogram excluded encephalic pathological activity.

**Figure 2:**
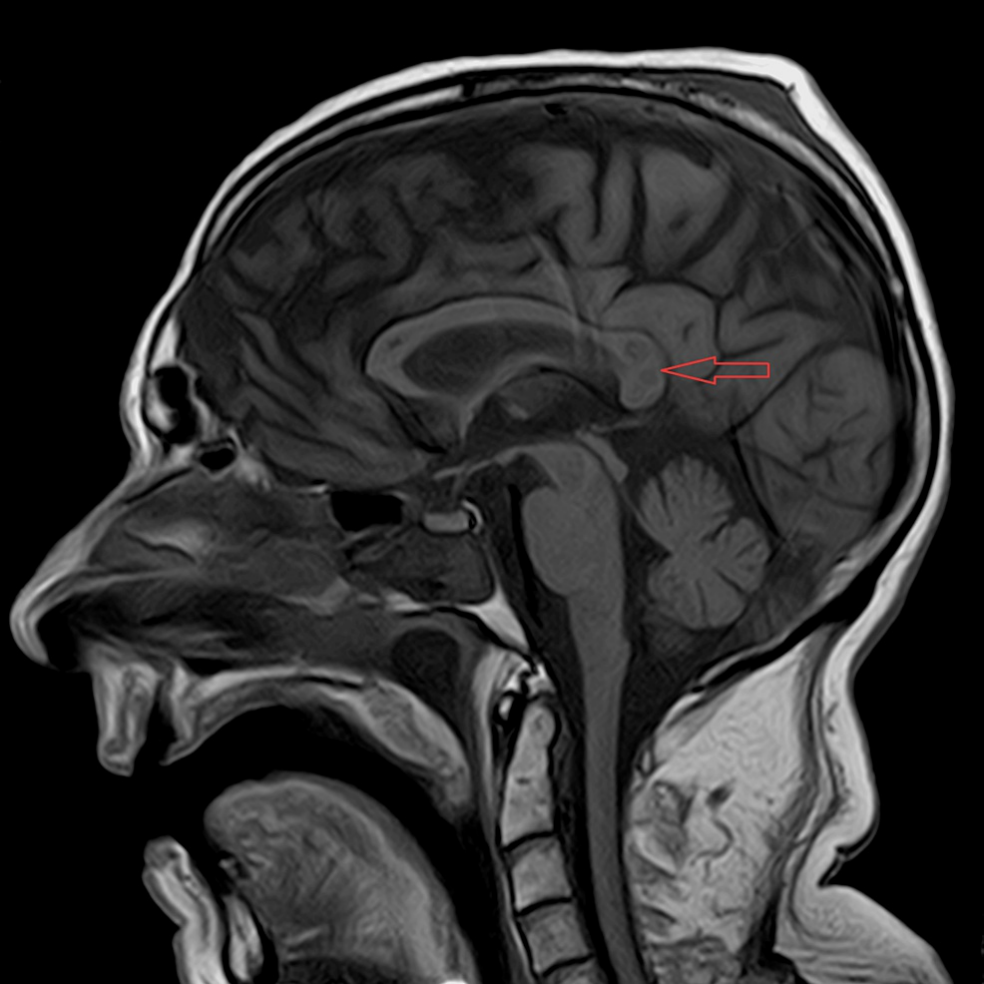
Sagittal T1 showing splenium lesions (arrow).

**Figure 3:**
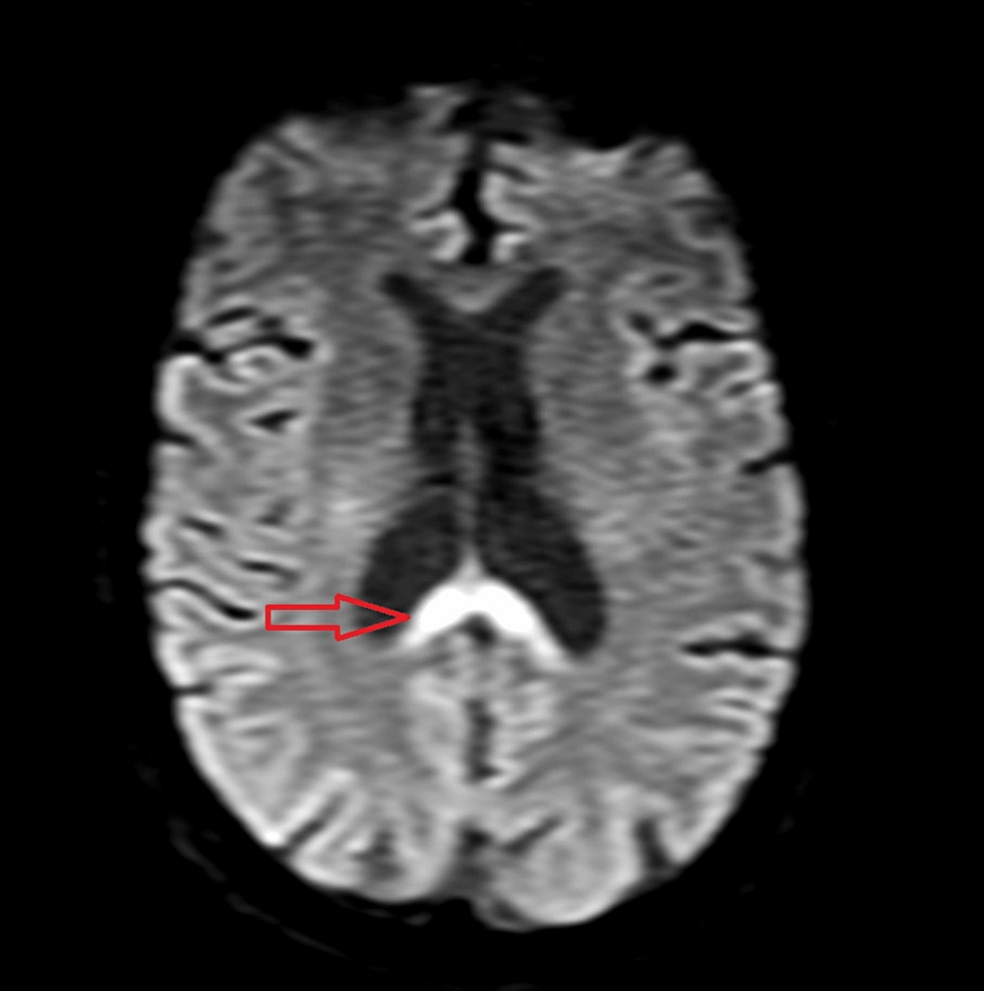
Axial DWI showing restriction to diffusion (arrow) which indicates an acute lesion. DWI: diffusion-weighted imaging

**Figure 4:**
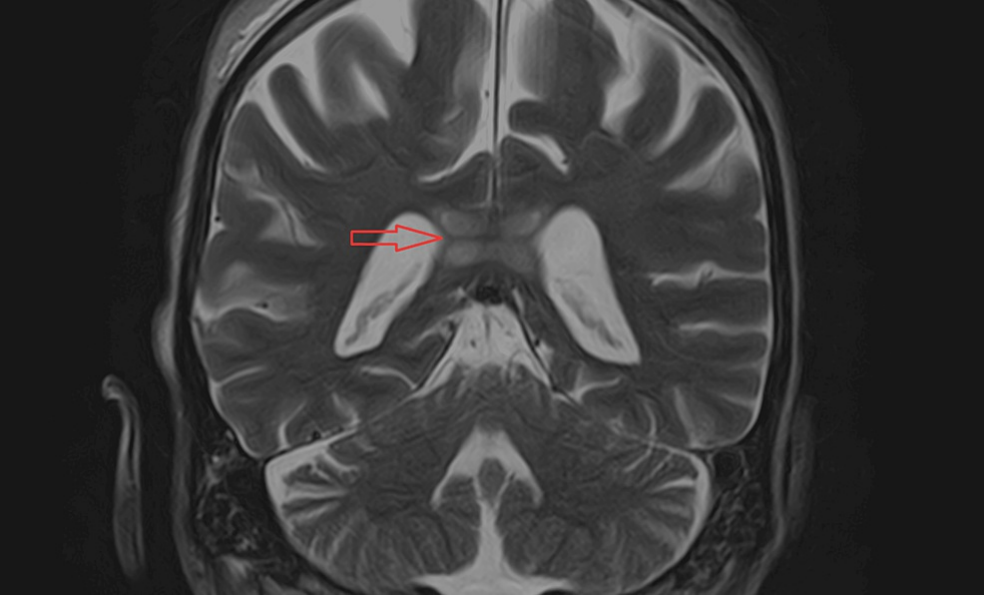
Coronal T2 showing hyperintense bilateral splenium lesions (arrow).

A cranial MRI confirmed the presence of lesions with central involvement of the genu and splenium of the corpus callosum and no extension to the cerebral hemispheres. The lesions showed edema and restricted diffusion (high signal in T2, fluid-attenuated inversion recovery (FLAIR), and diffusion-weighted imaging (DWI), and did not show any signal in apparent diffusion coefficient (ADC) map), indicating a recent lesion (Figures 2-4).

The imagiological features, along with the history of chronic alcohol abuse, were suggestive of MBD.

**Figure 5:**
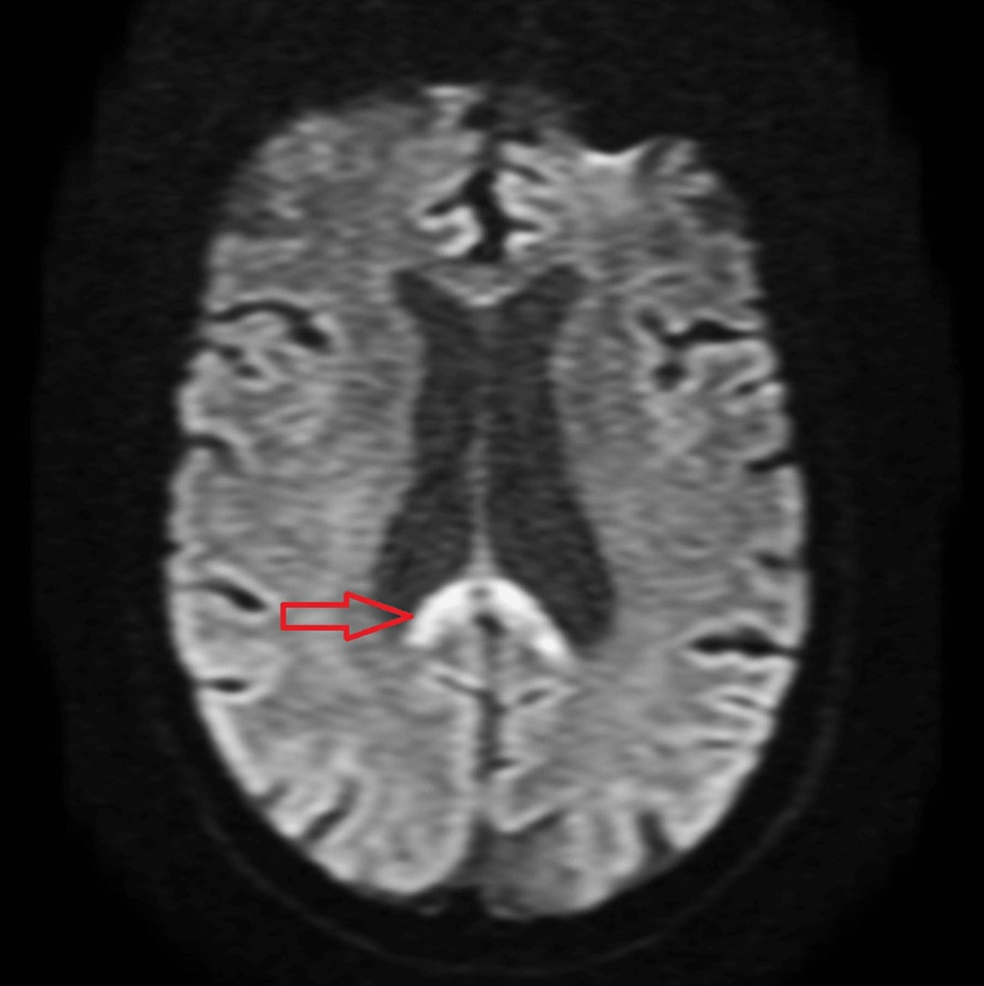
Axial DWI six months after the first MRI showing the attenuation of the restricted diffusion (arrow) compared to the initial MRI (Figure 3). DWI: diffusion-weighted imaging; MRI: magnetic resonance imaging

Treatment was started with thiamine 500 mg daily for 20 days, with an improvement in consciousness. He was extubated on the 17th ICU day. Significant recovery of motor functions was also noted. The patient was discharged from the hospital on the 24th day with scheduled appointments in Neurology, Psychiatry, and post-Intensive Care. Six months later he had stopped drinking. Complete recovery of motor functions was noted. Some confusion and memory loss, especially in household chores, remained. A depressed mood, frequent periods of anxiety, and death thoughts were present and were suggestive of post-traumatic stress disorder. A follow-up cranial MRI reevaluation showed the expected attenuation of the edema and restricted diffusion in the splenial lesions, indicating progression to chronicity (Figure 5).

## Discussion

We present a clinical case of a patient admitted to the hospital in a coma. He experienced worsening sensorium for some days prior to the ER assistance. He was admitted to the ICU and needed invasive mechanical ventilation. A medical history of chronic alcohol abuse, and malnourishment, along with the imagiological encephalic features, led us to consider a diagnosis of MBD. A high dose of thiamine was given with complete recovery.

Chronic alcohol use induces silent changes in the structure and function of the central and peripheral nervous systems that eventually result in irreversible, debilitating repercussions. The broader spectrum of alcohol-related neurologic dysfunction is complex, with pathophysiologic contributions from both direct effects of alcohol consumption, such as ethanol byproducts and reactive oxygen species accumulation, and indirect effects, such as thiamine deficiency and hepatic dysfunction [7].

A 1984 study [8], of hospitalized chronic alcoholics randomly selected at their first admission for detoxification, found 68% with cognitive deficits (aside from Wernicke encephalopathy), 74% with peripheral neuropathy, and 24% with evidence of autonomic dysregulation, all of which were presumed to be linked to chronic alcohol use. In adults, chronic alcohol abuse may lead to cerebral atrophy, dementia, cerebellar degeneration, osmotic demyelination, Morel’s laminar sclerosis, or MBD [9].

Attempts to classify MBD according to the course of the disease and the severity of the symptoms have been made. MBD was classically classified into acute, subacute, and chronic. Acute MBD included seizures, impairment of consciousness, and rapid death. Subacute MBD included variable degrees of mental confusion, dysarthria, behavioral abnormality, memory deficit, and impairment of gait. Chronic MBD, which is less common, was characterized by chronic dementia, which may progress over years [2].

More recently, Heinrich et al. [6] suggested a differentiation of MBD into only two subtypes, according to clinical and radiological characteristics: Type A, characterized by major impairment of consciousness, T2-hyperintense swelling of the entire corpus callosum on MRI, and poor outcome; Type B, with slight impairment of consciousness, and partial callosal lesions on MRI. Type B has a more favorable outcome. In roughly 50% of patients with a type A MBD, a prodromal stage with neuropsychiatric symptoms and gait disturbance has been reported, before the onset of coma. This could explain the initial symptoms presented by our patient, days before the ER admission.

Wernicke’s encephalopathy, delirium tremens, osmotic demyelination, or encephalitis may have very similar presentations and their features may overlap. This represents a difficulty in the differential diagnosis. The development of modern brain imaging techniques has allowed early detection of lesions suggestive of MBD, even in the absence of typical clinical syndromes. The classical MRI includes symmetric involvement of the corpus callosum with most commonly affection of the splenium, followed by the genu, and finally the body [1,5].

In some cases, the MRI lesions can be found in subcortical regions, cerebral lobes, hemispheric white matter, and basal ganglia. Such extra callosal lesions are primarily found in patients with poor prognoses and severe cognitive impairment. In contrast, patients with circumscribed lesions in the corpus callosum who receive an early diagnosis and appropriate treatment have a more favorable prognosis [5], as happened to our patient.

The impaired area has edematous changes with or without demyelination, which appears as a high signal lesion on T2, FLAIR, and DWI. As the acute stage passes, edematous changes gradually subside, and the high signal changes to a normal signal. However, if the disease progresses to permanent myelin impairment and necrosis, the MRI of the affected region shows atrophy and cystic transformation [1,10,11].

Hillbom et al. [1] reviewed 153 subjects with MBD. They reported that an acute altered conscience state was the most frequent presentation (80.4%). Loss of consciousness, dysarthria, impaired memory, signs of interhemispheric disconnection, and pyramidal signs were also frequently found. A significant linear trend for better outcomes among those patients treated with high-dose thiamine, especially when treated during the acute phase of the disease, was observed.

Corticosteroids were also proposed as a potential treatment for MBD. The rationale for this was a potential stabilization of the blood-brain barrier, a decrease in inflammatory edema, and suppression of leukocyte migration, especially lymphocytes. However, no clinical benefits of corticosteroids were ever shown [4,5].

## Conclusions

MBD is a rare condition with different, non-specific clinical presentations, and a challenging diagnosis. The MRI features can unveil suggestive lesions that, in an appropriate clinical context, may be diagnostic of MBD. Early diagnosis and effective treatment with high-dose thiamine are critical to improving prognosis. A strong suspicion, early neurological examination, and MRI are crucial to allow appropriate treatment and a better outcome.

## References

[1] Hillbom M, Saloheimo P, Fujioka S, Wszolek ZK, Juvela S, Leone MA (2014). Diagnosis and management of Marchiafava-Bignami disease: a review of CT/MRI confirmed cases. J Neurol Neurosurg Psychiatry.

[2] Tuntiyatorn L, Laothamatas J (2008). Acute Marchiafava-Bignami disease with callosal, cortical, and white matter involvement. Emerg Radiol.

[3] Hoshino Y, Ueno Y, Shimura H, Miyamoto N, Watanabe M, Hattori N, Urabe T (2013). Marchiafava-Bignami disease mimics motor neuron disease: case report. BMC Neurol.

[4] Singh S, Wagh V (2022). Marchiafava Bignami disease: a rare neurological complication of long-term alcohol abuse. Cureus.

[5] Shen YY, Zhou CG, Han N, Liang XM, Deng YQ (2019). Clinical and neuroradiological features of 15 patients diagnosed with Marchiafava-Bignami disease. Chin Med J (Engl).

[6] Heinrich A, Runge U, Khaw AV (2004). Clinicoradiologic subtypes of Marchiafava-Bignami disease. J Neurol.

[7] Hammoud N, Jimenez-Shahed J (2019). Chronic neurologic effects of alcohol. Clin Liver Dis.

[8] Franceschi M, Truci G, Comi G, Lozza L, Marchettini P, Galardi G, Smirne S (1984). Cognitive deficits and their relationship to other neurological complications in chronic alcoholic patients. J Neurol Neurosurg Psychiatry.

[9] Alderazi Y, Brett F (2007). Alcohol and the nervous system. Curr Diagn Pathol.

[10] Dong X, Bai C, Nao J (2018). Clinical and radiological features of Marchiafava-Bignami disease. Medicine (Baltimore).

[11] Kakkar C, Prakashini K, Polnaya A (2014). Acute Marchiafava-Bignami disease: clinical and serial MRI correlation. BMJ Case Rep.

